# Association of intellectual disability with violent and sexual crime and victimization: a population-based cohort study

**DOI:** 10.1017/S0033291722000460

**Published:** 2023-07

**Authors:** Antti Latvala, Magnus Tideman, Erik Søndenaa, Henrik Larsson, Agnieszka Butwicka, Seena Fazel, Paul Lichtenstein

**Affiliations:** 1 Institute of Criminology and Legal Policy, University of Helsinki, Helsinki, Finland; 2 Department of Medical Epidemiology and Biostatistics, Karolinska Institutet, Stockholm, Sweden; 3 School of Health and Welfare, Halmstad University, Halmstad, Sweden; 4 Forensic Department, St Olavs University Hospital, Trondheim, Norway; 5 School of Medical Sciences, Örebro University, Örebro, Sweden; 6 Child and Adolescent Psychiatry Stockholm, Stockholm Health Care Services, Region Stockholm, Sweden; 7 Department of Child Psychiatry, Medical University of Warsaw, Warsaw, Poland; 8 Department of Biostatistics and Translational Medicine, Medical University of Lodz, Lodz, Poland; 9 Department of Psychiatry, Warneford Hospital, University of Oxford, Oxford, UK

**Keywords:** Intellectual disability, violent offending, sexual offending, victimization, autism, ADHD

## Abstract

**Background:**

Intellectual disability (ID) is associated with violent and sexual offending and victimization, but the importance of neuropsychiatric comorbidity and severity of disability remains unclear.

**Methods:**

In a register-based cohort study of people born in Sweden 1980–1991 (*n* = 1 232 564), we investigated associations of mild and moderate/severe ID with any, violent and sexual crimes, and with assault victimization, stratified by comorbid autism and attention deficit hyperactivity disorder (ADHD). We defined ID by attendance at a special school or registered diagnosis and obtained data on criminal convictions and injuries or deaths due to assaults from nationwide registers until end of 2013.

**Results:**

Compared to people without ID, autism or ADHD, men and women with mild or moderate/severe ID and comorbid ADHD had elevated risks of violent crimes [range of hazard ratios (HRs) 4.4–10.4] and assault victimization (HRs 2.0–7.7). Women with mild ID without comorbidities or with comorbid autism also had elevated risks of violent crimes and victimization (HRs 1.8–4.6) compared to women without ID, autism or ADHD. The relative risks of sexual offending and victimization were elevated in men and women with ID without comorbidities (HRs 2.6–12.7). The highest risks for sexual offending in men (HRs 9.4–11.0) and for sexual assault victimization in women (HRs 11.0–17.1) related to ID and comorbid ADHD.

**Conclusions:**

The elevated risk of violent offending and assault victimization in people with ID is largely explained by comorbid ADHD, whereas ID is independently associated with sexual crimes and victimization, even though absolute risks are low.

## Introduction

There is a long-term interest in the risk of violence perpetration and victimization among people with intellectual disability (ID) (Holland, Clare, & Mukhopadhyay, 2002; Lindsay & Taylor, 2018; Simpson & Hogg, 2001). Challenging behavior is commonly observed in ID service settings (Holland et al., 2002), and while deinstitutionalization from the 1970s onwards resulted in individuals with ID successfully participating in the community, it also led to increased contacts with the criminal justice system due to cognitive and social vulnerabilities (Fisher, Baird, Currey, & Hodapp, 2016; Lindsay, Haut, & Steptoe, 2011). Early literature reported persons with ID being overrepresented in the offender population (Denkowski & Denkowski, 1985). Later studies have found that people with ID may be overrepresented among prisoners, but there is a large variation in the estimates (Fazel, Xenitidis, & Powell, 2008; Hellenbach, Karatzias, & Brown, 2017).

A handful of population-based studies have estimated the risk of crime and victimization in people with ID. Studies of disability service databases and population controls in Australia have found a threefold elevated risk of violent crimes and up to 16.5-fold risk of sexual offending, and similarly elevated risks of violent or sexual victimization (Fogden, Thomas, Daffern, & Ogloff, 2016; Nixon, Thomas, Daffern, & Ogloff, 2017; Thomas, Nixon, Ogloff, & Daffern, 2019). Register-based studies from Nordic countries have also found elevated risks of violent offending and other crimes (Hodgins, 1992; Moberg et al., 2015; Stevens, Laursen, Mortensen, Agerbo, & Dean, 2015). These studies, however, did not investigate victimization, included relatively small numbers of people with ID (*n* = 89–524), and used problematic definitions of ID, based solely on inpatient/outpatient diagnoses (Stevens et al., 2015), medical examination in connection with military conscription (Moberg et al., 2015), or placement in a special class in high school (Hodgins, 1992). The validity of the latter study has been questioned (Lindsay & Dernevik, 2013), but all these definitions are problematic as they are poorly validated and not comprehensive, leading to selected samples. A recent Swedish register-based study also found ID to be associated with an elevated risk of violent victimization in women (Ghirardi et al., 2021), but it defined ID only based on registered diagnoses from inpatient/outpatient treatment and did not separate mild *v*. more severe forms of ID.

It is also crucial to consider psychiatric comorbidities, which are very common in ID (Buckley et al., 2020; Cooper, Smiley, Morrison, Williamson, & Allan, 2007; Emerson & Hatton, 2007; Mazza, Rossetti, Crespi, & Clerici, 2020). Higher risks of offending and victimization have been found among people with ID and comorbid mental disorders as compared to those with ID without comorbidity (Fogden et al., 2016; Thomas et al., 2019). Among the most common comorbidities are the neurodevelopmental disorders of autism spectrum disorder (ASD) and attention deficit hyperactivity disorder (ADHD), with 8–30% of people with ID estimated to have ADHD, and likely even more with ASD, whose comorbidity may in part reflect diagnostic misclassification (Buckley et al., 2020; Emerson & Hatton, 2007; Thurm, Farmer, Salzman, Lord, & Bishop, 2019). Importantly, ASD and ADHD may be differentially associated with criminality: while ADHD is robustly associated with an increased risk of violent and non-violent offending, the association between ASD and crime may reflect comorbid conditions (Heeramun et al., 2017; Lundstrom et al., 2014; Mohr-Jensen, Bisgaard, Boldsen, & Steinhausen, 2019; Whiting, Lichtenstein, & Fazel, 2021).

Earlier studies of criminality and victimization have not separated mild and more severe forms of ID (Fogden et al., 2016; Hodgins, 1992; Moberg et al., 2015; Nixon et al., 2017; Stevens et al., 2015; Thomas et al., 2019), which are likely to differ in their associated risks. Taking into account the severity of ID is important from a clinical perspective, and because risk stratification will assist in resource allocation and preventative strategies.

There is a continuing need for knowledge about the risk of offending and victimization in people with ID, which can lead to better assessment and prevention of these outcomes. We conducted a nationwide register-based cohort study of the association of ID with criminal offending and assault victimization in Sweden, defining ID using data from special schools for people with ID in combination with register-based diagnoses of ID. We estimated the risks separately for people with ID with and without comorbid ASD and ADHD, and we compared the risks among those with mild *v.* moderate/severe ID.

## Methods

### Study population

We linked several Swedish registers using a unique personal identity number given to all citizens at birth. Follow-up data were available until the end of 2013. We defined ID using data from the Halmstad University Register on Pupils with Intellectual Disability (HURPID) (Arvidsson, Widen, & Tideman, 2015) in combination with inpatient and outpatient diagnoses from the National Patient Register (NPR) (Ludvigsson et al., 2011). HURPID data included individuals born 1980–1991; thus we selected people born in Sweden during that period from the Total Population Register (Ludvigsson et al., 2016) as the study population. Information about emigrations and deaths was available from the Migration Register and the Cause of Death Register (Brooke et al., 2017), respectively. Using the Multi-Generation Register (Ekbom, 2011), we linked individuals with their parents, enabling the identification of siblings in the study population. Requiring non-missing data for both parents resulted in an analysis sample of 1 232 564 individuals (51% men). HURPID has been approved by the Ethical Review Board in Lund, and the register linkages for the current study were approved by the Regional Ethical Review Board of Stockholm.

### Exposure and outcome measures

#### Mild and moderate/severe ID

HURPID includes individuals who graduated in 2001–2011 from a special upper secondary school for pupils with intellectual disabilities in Sweden. The Special Secondary School curriculum offers different educational programs, including national programs, which have a common curriculum, vocational programs, programs specific to particular schools, and tailored individual training programs. Individuals who attend a special secondary school do not necessarily have a medical diagnosis of ID, but have been assessed to have ID at the school health care system. Students with mild intellectual disabilities usually attend either a national program or a school-specific program, and those with more severe disabilities are more likely to attend individualized programs (Arvidsson et al., 2015). Thus, we classified individuals who had graduated from training programs as having moderate/severe ID (*n* = 1052), whereas individuals who had graduated from the other types of programs were classified as having mild ID (*n* = 9893).

Because HURPID does not include all individuals with ID in Sweden, we also included ID as defined by the International Classification of Diseases (ICD) revision eight (years 1980–1986), nine (1987–1996) and ten (1997–2013) diagnoses in the NPR (diagnostic codes given in online Supplementary Table S1). We separated mild ID (*n* = 5562) from the other ID diagnoses, which were classified as moderate/severe ID (*n* = 4782).

For a combined variable for ID to be used in the analyses, we prioritized the more severe classification. Thus, we classified those who had mild ID according to HURPID, NPR, or both, and did not have moderate/severe ID in either data source as having mild ID [*n* = 11 689 of which 6777 (58.0%) men, 4912 women]. Those who had moderate/severe ID in HURPID, NPR, or both were classified as having moderate/severe ID [*n* = 5166 of which 3003 (58.1%) men, 2163 women]. A cross-tabulation of ID in HURPID and NPR is given in online Supplementary Table S2.

#### Criminality

We included data on criminal convictions from the National Crime Register at age 15 (age of criminal responsibility) and older. We studied any crimes, violent crimes (excluding sexual crimes), and sexual crimes separately. The included criminal offenses are listed in online Supplementary Table S1. In sensitivity analyses, we studied suspicions of crimes, available from the Register of People Suspected of Offences. Similar to criminal convictions, we studied any criminal suspicions, violent crime suspicions, and sexual crime suspicions separately.

#### Violent victimization

We defined violent victimization as inpatient or outpatient diagnoses of injuries due to assaults in the NPR, and as deaths due to assaults, using the same ICD codes (online Supplementary Table S1), in the Cause of Death Register (Brooke et al., 2017). We also studied sexual victimization separately, defined as ICD diagnoses of sexual assault and abuse, including rape, in the NPR.

### Covariates

We included parental education as an indicator of the family's socioeconomic status, defined as the highest attained education of either parent, available from National Census data for years 1970–1985 and the Longitudinal Integration Database for Health Insurance and Labour Market Studies (LISA) for 1990–2013. We also included parents' immigrant status as a dichotomous variable denoting whether at least one parent was not born in Sweden. To adjust for potential cohort and period effects, we included birth year. We stratified the analyses by comorbid ASD and ADHD. ASD was defined as a diagnosis in the NPR at any time during the study period, and ADHD was defined by having a diagnosis in the NPR or ever been dispensed a drug used almost exclusively in the treatment of ADHD (online Supplementary Table S1), based on data from the Swedish Prescribed Drug Register (PDR) for 2005–2013 (Larsson et al., 2013; Wettermark et al., 2007). The diagnostic accuracy of ASD in Swedish registers, with 96% of diagnoses found consistent in a case note validation study, as well as ADHD identified either through NPR or PDR has been shown to be good (Idring et al., 2012; Larsson et al., 2013).

### Statistical methods

#### Main analyses

We fitted Cox proportional hazard regression models to estimate the relative hazard of criminal offending and assault victimization during the follow-up in individuals with mild or moderate/severe ID as compared to those without ID. We derived separate estimates for (1) people with ID but no ASD or ADHD, (2) people with ID and ASD but no ADHD, and (3) people with ID and ADHD but no ASD, in each case having people without ID, ASD and ADHD as the reference category. We assessed the role of ID severity and neuropsychiatric comorbidity for the relative risks by inspecting overlap in the point estimates and their 95% confidence intervals between the different subcategories of people with ID. We conducted all analyses separately for men and women.

We conducted separate analyses for any criminal convictions, violent crime convictions, and sexual crime convictions as outcomes. Similarly, we conducted Cox regression models for any assault victimization and for sexual assault victimization separately. In the models for criminal convictions, the participants were followed from age 15 until the occurrence of the first respective conviction, and in the models for ID and assault victimization, the follow-up was from birth until the first registered assault victimization. As ID is generally a congenital condition, and the timing of ID registration does not correspond with the onset of disability but rather the first available date of contact with specialist healthcare services or special school attendance in our data, we treated people with ID as exposed from birth onwards. Those who had no registrations of convictions or assaults during the study period in the corresponding analyses contributed person-time at risk until the end of follow-up (31 December 2013), date of emigration, or death, whichever occurred first. We adjusted all analyses for birth year (as a categorical variable), parental education, and parental immigration status. We inspected the Schoenfeld residuals graphically and did not observe violations of the proportional hazards assumption.

#### Sensitivity analyses

To inspect potential biases in the judicial process, we studied criminal suspicions by the police as outcome variables, following participants from age 15 until the first suspicion. To address possible familial (i.e. genetic and shared environmental) effects for the associations of ID with offending and victimization, we repeated all analyses conducting stratified Cox regression analyses within clusters of full brothers and sisters (Allison, 2009). As further sensitivity analyses, we conducted the population-level analyses separately for ID defined based on the HURPID and NPR data, and in order to rule out potential reverse causality for the association between ID and assault victimization, we repeated the main analysis excluding those with assault victimization predating ID registration. Finally, to complement the main analyses, we also conducted the analyses for those with ID and both ASD and ADHD.

## Results

Descriptive data of people with and without ID are presented in Table 1. Of people with mild ID, 14.2% had ASD and 17.3% had ADHD, and the corresponding proportions were 27.8 and 10.4% among people with moderate/severe ID. Further, 26.9% of people with mild ID and 33.9% of those with moderate/severe ID had either ASD or ADHD. Online Supplementary Table S3 gives the person-years at risk and incidence rates for criminal convictions and assault victimization.

**Table 1.** Characteristics of the study population (*N* = 1 232 564)

Men	No ID *N* = 623 401 (98.46%)	Mild ID *N* = 6777 (1.07%)	Moderate/severe ID *N* = 3003 (0.47%)
Birth year, Median (quartiles)	1986 (1983, 1989)	1987 (1984, 1989)	1987 (1983, 1989)
Parental education, No. (%)			
High	292 886 (47.0)	1525 (22.5)	1010 (33.6)
Middle	294 137 (47.2)	4366 (64.4)	1685 (56.1)
Low	34 242 (5.5)	882 (13.0)	300 (10.0)
Missing	2136 (0.3)	4 (0.06)	8 (0.3)
Parental immigration, No. (%)	45 570 (7.3)	477 (7.0)	301 (10.0)
ASD, No. (%)	6960 (1.1)	1111 (16.4)	937 (31.2)
ADHD, No. (%)	17 434 (2.8)	1380 (20.4)	370 (12.3)
Any criminal conviction, No. (%)	139 382 (22.4)	1810 (26.7)	272 (9.1)
Violent criminal conviction, No. (%)	37 684 (6.0)	710 (10.5)	145 (4.8)
Sexual criminal conviction, No. (%)	2040 (0.3)	137 (2.0)	20 (0.7)
Any assault victimization, No. (%)	33 692 (5.4)	408 (6.0)	64 (2.1)
Sexual assault victimization, No. (%)	70 (0.01)	12 (0.2)	3 (0.1)
Age at ID registration, Mean (s.d.)	–	18.9 (4.1)	12.2 (7.5)
Age at first criminal conviction, Mean (s.d.)	19.3 (3.6)	19.3 (3.3)	19.7 (3.9)
Age at first violent crime conviction, Mean (s.d.)	19.9 (3.6)	20.2 (3.6)	20.9 (3.9)
Age at first sexual crime conviction, Mean (s.d.)	21.7 (4.1)	20.7 (3.6)	21.5 (3.2)
Age at first assault victimization, Mean (s.d.)	21.4 (3.7)	21.0 (4.2)	20.8 (3.7)
Age at first sexual assault victimization, Mean (s.d.)	18.3 (4.8)	15.2 (5.5)	22.7 (5.1)
Women	No ID *N* = 592 308 (98.82%)	Mild ID *N* = 4912 (0.82%)	Moderate/severe ID *N* = 2163 (0.36%)
Birth year, Median (quartiles)	1986 (1983, 1989)	1987 (1984, 1989)	1987 (1983, 1989)
Parental education, No. (%)			
High	277 539 (46.9)	1123 (22.9)	763 (35.3)
Middle	279 742 (47.2)	3131 (63.7)	1180 (54.6)
Low	32 940 (5.6)	656 (13.4)	218 (10.1)
Missing	2087 (0.4)	2 (0.04)	2 (0.1)
Parental immigration, No. (%)	43 426 (7.3)	350 (7.1)	197 (9.1)
ASD, No. (%)	3915 (0.7)	553 (11.3)	500 (23.1)
ADHD, No. (%)	12 102 (2.0)	644 (13.1)	167 (7.8)
Any criminal conviction, No. (%)	53 246 (9.0)	566 (11.5)	103 (4.8)
Violent criminal conviction, No. (%)	7248 (1.2)	172 (3.5)	42 (1.9)
Sexual criminal conviction, No. (%)	31 (0.01)	3 (0.06)	–
Any assault victimization, No. (%)	12 021 (2.0)	334 (6.8)	69 (3.2)
Sexual assault victimization, No. (%)	4769 (0.8)	245 (5.0)	51 (2.4)
Age at ID registration, Mean (s.d.)	–	19.2 (4.1)	12.4 (7.7)
Age at first criminal conviction, Mean (s.d.)	18.8 (3.4)	19.6 (3.6)	19.9 (4.0)
Age at first violent crime conviction, Mean (s.d.)	19.7 (3.8)	20.4 (3.7)	–
Age at first sexual crime conviction, Mean (s.d.)	21.1 (3.8)	23.4 (2.9)	21.5 (3.2)
Age at first assault victimization, Mean (s.d.)	20.9 (4.2)	20.6 (4.0)	20.8 (5.4)
Age at first sexual assault victimization, Mean (s.d.)	20.0 (3.9)	20.3 (4.1)	20.3 (5.4)

ID, intellectual disability; ASD, autism spectrum disorder; ADHD, attention deficit hyperactivity disorder.

### Association of ID with offending and assault victimization

**Fig. 1.**
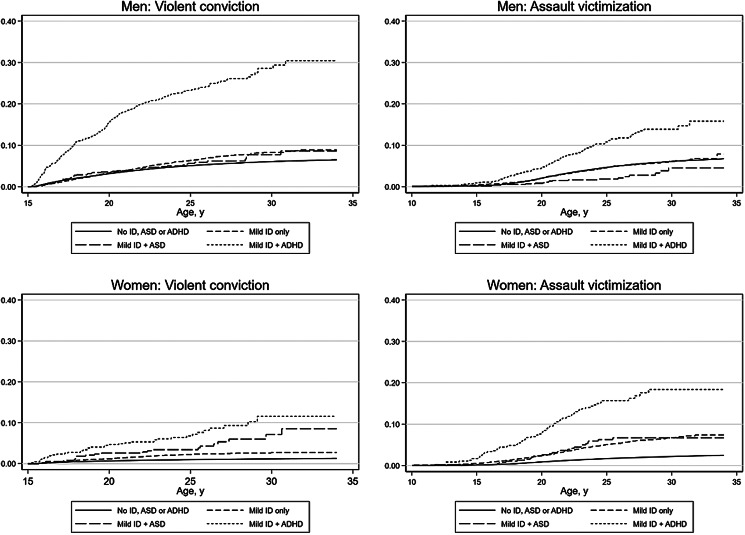
Cumulative incidence (estimated as 1 – the Kaplan-Meier estimate of the survival function under the assumption of no competing risks) of violent criminal convictions and any assault victimization in men and women with mild ID, stratified by comorbid ASD and ADHD.

Figure 1 shows the cumulative incidence of violent convictions and assault victimization in men and women with mild ID, stratified by comorbid ASD and ADHD. The cumulative incidence of violent convictions by age 34 (maximum age at the end of follow-up) was markedly elevated in men with mild ID and ADHD [30.4% (95% CI 26.3–35.1%)] compared to men with mild ID without comorbidities [8.9% (7.9–10.1%)]. Similarly, women with mild ID and ADHD [11.6% (7.9–16.9%)] as well as mild ID and ASD [8.5% (5.0–14.3%)] had a higher cumulative incidence of violent convictions than did women with mild ID without comorbidities [2.7% (2.2–3.4%)]. The cumulative incidence of sexual crime convictions in men and sexual assault victimization in women with mild ID are shown in online Supplementary Fig. S1. The cumulative incidence estimates for all outcomes and groups are given in online Supplementary Tables S4 and S5.

**Fig. 2.**
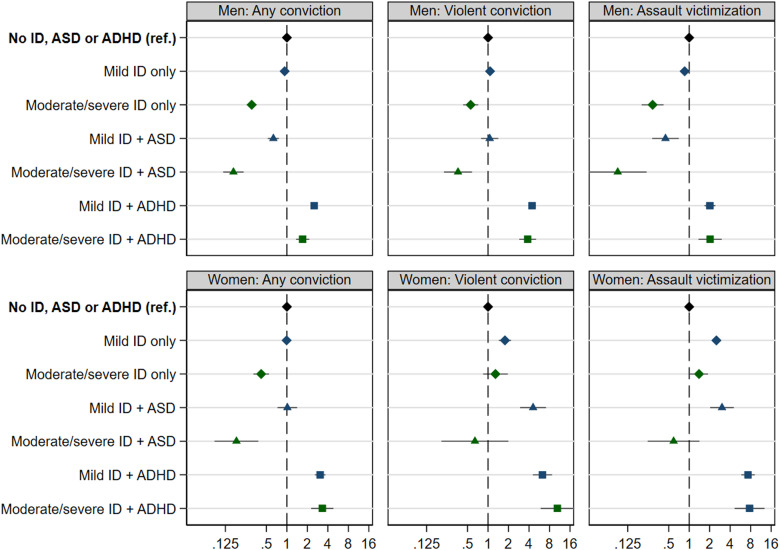
Cox proportional hazard ratios (with 95% CIs) for any criminal convictions, violent criminal convictions, and any assault victimization associated with mild (blue markers) and moderate/severe ID (green markers), stratified by comorbid ASD (triangles) and ADHD (squares). Please note the logarithmic scale.

Results from Cox models for any convictions, violent convictions and assault victimization are shown in Fig. 2. In men, ID was only associated with elevated risks for these outcomes in combination with ADHD. Men with mild or moderate/severe ID and ADHD had 1.7–4.4 fold elevated risk as compared to those without ID and ADHD, whereas risks were reduced or unaffected in men with ID without comorbidities [hazard ratios (HRs) 0.3–1.1] (online Supplementary Table S6). In women, mild ID without comorbid ASD or ADHD was associated with a 1.8–2.5 fold risk for violent convictions and assault victimization. Risks were higher or similar in women with mild ID and ASD (HRs 3.0–4.6) and higher in women with ID and ADHD (HRs 6.3–10.4) (online Supplementary Table S6).

**Fig. 3.**
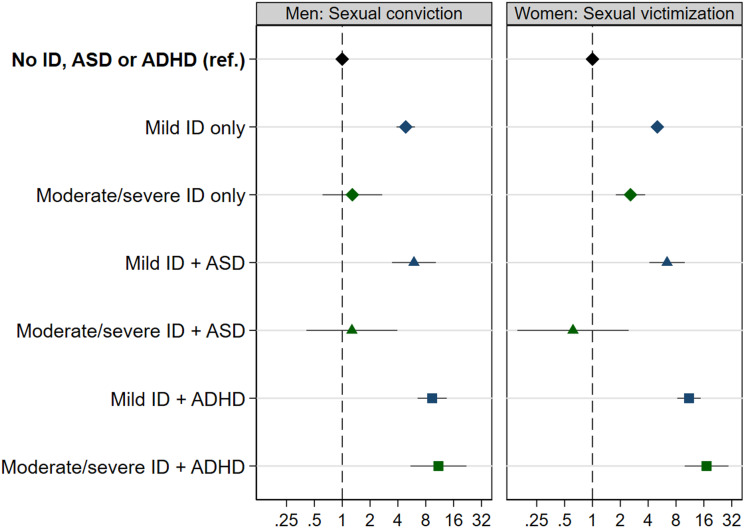
Cox proportional hazard ratios (with 95% CIs) for sexual crime convictions in men and sexual assault victimization in women associated with mild (blue markers) and moderate/severe ID (green markers), stratified by comorbid ASD (triangles) and ADHD (squares). Please note the logarithmic scale.

The associations of ID with sexual crime convictions in men and with sexual assault victimization in women are displayed in Fig. 3. Both with and without comorbidities, mild ID was associated with sexual offending, with the strongest association among men with comorbid ADHD (HR = 9.39, 95% CI 6.54–13.5). The estimate for men with moderate/severe ID and comorbid ADHD was elevated but imprecise. The risk of sexual crimes was also elevated in women with mild ID without comorbidities (online Supplementary Table S7).

With the exception of moderate/severe ID with ASD, women with ID had an elevated risk of sexual assault victimization, with the highest risks among those with comorbid ADHD (HR 11.0–17.1). The risk of sexual victimization was also elevated in men with ID, but the estimates were imprecise (online Supplementary Table S7).

### Sensitivity analyses

Associations of ID with criminal suspicions were generally similar to those with convictions (online Supplementary Table S8). In within-family analyses, associations were attenuated, but there remained elevated risks of sexual crimes in men and sexual victimization in women (online Supplementary Tables S8–S10). The risks of criminality and assault victimization were consistently higher among those with a diagnosis of ID as compared to those having graduated from a special school (online Supplementary Table S11). When excluding people with assault victimization predating ID registration (*n* = 333), the associations between ID and victimization were often attenuated but elevated risks remained especially for sexual assault victimization among women with ID (online Supplementary Table S12). Finally, the relative risks among those with ID and both ASD and ADHD were generally between the risks seen in people with ID and either ASD or ADHD (online Supplementary Table S13).

## Discussion

In this large population-based cohort study, we found the associations of ID with violent and sexual offending and with assault victimization to vary substantially by the comorbidity with neurodevelopmental disorders and by the severity of ID. Specifically, the risks of violence perpetration and victimization were highly elevated in people with ID who had co-occurring ADHD. In contrast, while the highest risk for sexual offending and victimization was observed in people with ID and comorbid ADHD, ID was associated with these outcomes even without comorbidities.

Two other findings are notable. The first is the high rates of comorbidities at 17% for ADHD and 14% for ASD in those with mild ID. The second is the generally low absolute rates of the outcomes, especially among people with ID without comorbidities. Less than 1 in 10 people with ID had violent convictions and assault victimization by age 34, and about 3% of men with ID had sexual convictions.

Overall, our results were consistent with earlier findings (Fogden et al., 2016; Moberg et al., 2015; Nixon et al., 2017; Stevens et al., 2015; Thomas et al., 2019). We found higher relative risks for sexual offending and sexual victimization as compared to violent crimes and any victimization, as has been found earlier. The magnitude of the relative risk estimates was also similar to those reported previously. However, our findings extend the literature in important ways. First, our sample of people with ID is several times larger than the previous studies, adding precision to the findings. With the register-based design, we were also able to compare risks to the general population, which is novel especially for sexual offending. Second, our definition of ID, based on data from special schools and clinical diagnoses of ID, is likely to capture ID better in the general population than the more restricted definitions used in some earlier studies. Third, we were able to study the contributions of ID severity and neuropsychiatric comorbidity, which were highly relevant for the associations.

Our findings have clinical relevance. The elevated risk for both sexual offending and victimization suggests a need for specific prevention and intervention efforts. Importantly, ID was strongly associated with sexual offending and victimization among siblings discordant for ID, suggesting that familial confounding did not explain the associations. Focusing on factors such as attachment, intimacy, sexual knowledge and interests, cognitive distortions, and social skills has been suggested, and some research support exists for learning strategies to respond to problematic situations and developing adaptive skills and decision-making abilities, combined with individually tailored support (Jones, 2007).

An understanding of why the risks of sexual offending and victimization are specifically elevated is crucial for successful risk reduction. Several factors likely to contribute to problematic sexual behavior among men with ID have been discussed, and different theoretical perspectives such as access to sexually related information and education, generic male sexual violence, the sexuality of people with ID, and challenging behavior among people with ID more generally can be employed (Martinello, 2015; Medina-Rico, López-Ramos, & Quiñonez, 2018; Thompson & Brown, 1997). People with ID have the same sexual needs as the general population, but are more vulnerable, with a greater risk of sexual abuse and problematic sexual behavior. A review of the literature concludes that the biggest challenge for a healthy sexuality in people with ID is the lack of accessible information and sexual education to support sound relations (Medina-Rico et al., 2018). Especially educational interventions and a supported network may have positive effects. The elevated risk of sexual offending could be partly explained even if there were no differences in sexuality between men with and without ID. Men with ID could be less successful in keeping their sexual behavior covert compared to other men due to unawareness of conventions related to privacy or relative naivety and honesty in reporting their sexual behavior (Thompson & Brown, 1997). Men with ID are also likely to have a more limited understanding of the social significance of their sexual behavior, and they may lack intimate relationships and have therefore less acceptable opportunities for sexual expression. Men with ID are also more likely to have experienced sexual abuse themselves, which may increase their risk of sexual offending (Balogh et al., 2001; Thompson, 1997). On the other hand, it is possible that men with ID have differences in sexual interests compared to men without ID. For example, men with ID may less selective in their choice of victims (Thompson, 1997; Thompson & Brown, 1997). Finally, there may be specific ID-related neurobiological deficits leading to problems with impulse control and disinhibited sexual behavior, which could increase the risk of both sexual offending and victimization.

It would also be important to understand the specific mechanisms underlying the risk of sexual victimization in people with ID. In our results, there was a striking similarity in the patterns of risk for sexual offending in men and sexual victimization in women. Prior studies suggest that it is common for both the victim and the perpetrator to have ID (Furey & Niesen, 1994; McCarthy & Thompson, 1997; Petersilia, 2001), and that people with ID are often both victims and perpetrators of sexual abuse (Balogh et al., 2001). While our data did not enable linking victims and perpetrators, it is likely that overlap with regard to ID exists. Besides other people with ID, caregivers and family members have been indicated as potential perpetrators of people with ID (Petersilia, 2001).

Our findings underline the importance of assessing neuropsychiatric comorbidities in ID (Thurm et al., 2019), and where possible their treatment. ADHD has been consistently found to be associated with violent and non-violent offending (Lundstrom et al., 2014; Mohr-Jensen et al., 2019; Whiting et al., 2021) as well as with violent and sexual victimization (Ghirardi et al., 2021; Snyder, 2015, 2019). In contrast, ASD may not be independently associated with offending but may increase the risk of violent and sexual victimization, especially in women (Brown-Lavoie, Viecili, & Weiss, 2014; Ghirardi et al., 2021; Lundstrom et al., 2014; Weiss & Fardella, 2018; Whiting et al., 2021). ID with comorbid ADHD was associated with the highest risk for offending and victimization in the current study. In addition, women with mild ID and comorbid ASD had a higher relative risk of violent convictions as compared to women with mild ID without comorbidities. However, ID without comorbidities was associated with violent offending and victimization in women but not in men. Reasons for this difference are unclear but may include more sub-threshold ADHD or undiagnosed ASD in women with ID. The finding is consistent with a recent register-based study, which used a partly overlapping sample but a different analytic strategy. This previous study reported that women, but not men, with ID defined as a time-varying exposure based on diagnoses in the NPR had an elevated risk for violent victimization even adjusting for ASD and ADHD (Ghirardi et al., 2021). Regardless of the mechanism, the elevated risk for violence and victimization in men and women with ID who also have ADHD, and the risk for violence in women with ID who also have ASD should be recognized in ID services and interventions considered to reduce risk. These may include medication in ADHD (Lichtenstein et al., 2012) and psychosocial supports in ASD (Pallathra, Cordero, Wong, & Brodkin, 2019).

Several important factors and limitations related to our study should be considered. First, officially recorded crimes only capture a fraction of all criminal behavior, and like all register-based studies, this one is subject to that limitation. Second, the study of offending in people with ID can be specifically hampered by biases due to differential treatment in the criminal process all the way from reporting of suspected crimes up to court convictions (Holland et al., 2002). We were able to investigate this by comparing the associations of ID with court convictions to those with suspicions by the police. Overall, there was no clear pattern of differences between the two data sources, suggesting no systematic bias in the treatment of people with ID in courts in Sweden as compared to police investigation (including processes leading to investigation), but we were unable to assess potential police bias. We also adjusted the analyses for parental education (which is a proxy for socioeconomic status) and immigration, which may affect the registration of ID as well as handling in the criminal process. In addition, due to the nature of the data used in this study, the estimated associations of ID with violence perpetration and victimization may suffer from both upward and downward biases. For example, as the follow-up did not continue beyond young adulthood, we do not fully capture forms of violence that are more common at older ages, such as intimate partner violence. On the other hand, as follow-up for criminality started only at age 15, we do not capture offending among youth and thus underestimate the prevalence of sexual and non-sexual violent crimes. We may also over- or underestimate the association between ID and criminality because we are more likely to capture those with more persistent offending compared to those who desist before age 15. Further, as our measures of victimization rely on medical treatment, we may underestimate victimization among people with ID who have communication limitations, which may affect the likelihood of seeking help and being treated. Of note, there was heterogeneity in our definition of ID, and the associations with offending and victimization were consistently stronger among those with a diagnosis of ID in the NPR as compared to those having graduated from a special school. When the two data sources disagreed, we prioritized the more severe classification, which likely had an effect on our results. Finally, men with sexual offenses against children differ from men with sexual offenses against adults in various characteristics, including risk factors for offending. By treating sexual offending as a single category, we may have missed important differences in the offending profiles between men with and without ID. An important goal for future research would be to analyze in more detail the patterns and types of sexual and non-sexual violent offending among people with ID.

## Conclusions

Our findings suggest the association of ID with violent offending and victimization to be largely explained by comorbid ADHD. In contrast, the risk of sexual offending and victimization is clearly elevated in people with ID independently of neuropsychiatric comorbidity. More research is required to address what types of interventions are effective in young people and adults with ID, especially those with ADHD. There is a need for collaboration between the social services, schools, disability services, the health care system, the police, the criminal justice system as well as people with ID and their networks. Addressing the complex needs of this population remains a substantial challenge.

## Supplementary material

For supplementary material accompanying this paper visit https://doi.org/10.1017/S0033291722000460. click here to view supplementary material
