# Supplementary material for: Association of intellectual disability with violent and sexual crime and victimization: a population-based cohort study
Source: Psychol Med. 2022 Mar 3;53(9):3817–25. doi: 10.1017/S0033291722000460 (PMC10317804; doi:10.1017/S0033291722000460)
Supplement: Supplementary file 1 [file S0033291722000460sup001.docx]

**Supplementary Material for Latvala *et al.* Association of Intellectual Disability with Violent and Sexual Crime and Victimization: A Population-Based Cohort Study**

**Supplementary Table 1.** International Classification of Diseases (ICD) and Anatomical Therapeutic Chemical (ATC) codes used to define ID, ASD, ADHD, and assault victimization (top), and offenses included in violent and sexual crimes (bottom)

|  | **ICD-8** | **ICD-9** | **ICD-10** | **ATC** |
| --- | --- | --- | --- | --- |
| Mild ID | 311 | 317 | F70 |  |
| Moderate/severe ID | 312–315 | 318, 319 | F71–F73, F78, F79 |  |
| ASD |  | 299 | F84.0, F84.1, F84.3, F84.5, F84.8, F84.9 |  |
| ADHD |  | 314 | F90 | N06BA04, N06BA01, N06BA02, N06BA09, NO6BA12 |
| Any assault victimization | E960–E969 | E960–E969 | X85–Y09 |  |
| Sexual assault victimization |  | E960.1 | Y05, T74.2 |  |
|  | **Criminal convictions** | | | |
| Violent crime | Murder, manslaughter, assault, kidnapping, illegal restraint, illegal coercion or threats, robbery, threats or violence against an officer, arson, gross violation of a person’s integrity, harassment | | | |
| Sexual crime | Rape, sexual coercion, child molestation, sexual intercourse with a child, child pornography offenses, pimping, sexual harassment | | | |

ID, intellectual disability; ASD, autism spectrum disorder; ADHD, attention deficit hyperactivity disorder

**Supplementary Table 2.** The agreement of ID variables in the HURPID and NPR data

|  | HURPID data | | | |
| --- | --- | --- | --- | --- |
| NPR data | **No ID** | **Mild ID** | **Moderate/severe ID** | **Total** |
| **No ID**  *Row %*  *Column %* | 1,215,709  *99.5*  *99.5* | 6181  *.5*  *62.5* | 330  *.03*  *31.4* | 1,222,220  *100.0*  *99.2* |
| **Mild ID**  *Row %*  *Column %* | 3046  *54.8*  *.3* | 2462  *44.3*  *24.5* | 54  *1.0*  *5.1* | 5562  *100.0*  *.5* |
| **Moderate/Severe ID**  *Row %*  *Column %* | 2864  *59.9*  *.2* | 1250  *26.1*  *12.6* | 668  *14.0*  *63.5* | 4782  *100.0*  *.4* |
| **Total**  *Row %*  *Column %* | 1,221,619  *99.1*  *100.0* | 9893  *.8*  *100.0* | 1052  *.1*  *100.0* | 1,232,564  *100.0*  *100.0* |

ID, intellectual disability; HURPID, Halmstad University Register on Pupils with Intellectual Disability; NPR, National Patient Register

**Supplementary Table 3.** Person-years at risk and rates (95% confidence intervals) of criminal convictions and assault victimization in people with no ID, mild ID, and moderate/severe ID

|  | **No ID** | **Mild ID** | **Moderate/severe ID** |
| --- | --- | --- | --- |
| **Men** |  |  |  |
| Any criminal conviction |  |  |  |
| Person-years at risk | 6,331,565 | 65,663 | 31,744 |
| Rate (95% CI) per 10,000 person-years | 216.3 (215.2, 217. 5) | 272.9 (260.6, 285.8) | 83.5 (74.0, 94.2) |
| Violent crime conviction |  |  |  |
| Person-years at risk | 7,209,979 | 74,553 | 32,919 |
| Rate (95% CI) per 10,000 person-years | 51.3 (50.8, 51.8) | 93.9 (87.2, 101.1) | 43.1 (36.6, 50.8) |
| Sexual crime conviction |  |  |  |
| Person-years at risk | 7,486,650 | 78,398 | 33,815 |
| Rate (95% CI) per 10,000 person-years | 2.67 (2.56, 2.79) | 17.3 (14.7, 20.5) | 5.91 (3.82, 9.17) |
| Assault victimization |  |  |  |
| Person-years at risk | 16,459,247 | 178,211 | 77,386 |
| Rate (95% CI) per 10,000 person-years | 20.5 (20.3, 20.7) | 22.9 (20.8, 25.2) | 8.27 (6.47, 10.6) |
| **Women** |  |  |  |
| Any criminal conviction |  |  |  |
| Person-years at risk | 6,611,586 | 53,586 | 23,213 |
| Rate (95% CI) per 10,000 person-years | 79.1 (78.4, 79.8) | 105.1 (96.7, 114.1) | 43.5 (35.8, 52.9) |
| Violent crime conviction |  |  |  |
| Person-years at risk | 7,022,692 | 56,557 | 23,706 |
| Rate (95% CI) per 10,000 person-years | 10.1 (9.9, 10.4) | 30.2 (26.0, 35.1) | 17.7 (13.1, 24.0) |
| Sexual crime conviction |  |  |  |
| Person-years at risk | 7077487 | 57700 | 24001 |
| Rate (95% CI) per 10,000 person-years | .04. (.03, .06) | .52 (.17, 1.61) | 0 |
| Assault victimization |  |  |  |
| Person-years at risk | 15,707,061 | 129,111 | 55,049 |
| Rate (95% CI) per 10,000 person-years | 7.65 (7.52, 7.79) | 25.9 (23.2, 28.8) | 12.5 (9.90, 15.9) |

ID, intellectual disability

**Supplementary Table 4.** Cumulative incidence (95% confidence interval) of any criminal convictions, violent criminal convictions, and any assault victimization by age 34 in people with mild and moderate/severe ID, stratified by comorbid ASD and ADHD

|  | **Any conviction** | | **Violent conviction** | | **Any assault** | |
| --- | --- | --- | --- | --- | --- | --- |
|  | Men | Women | Men | Women | Men | Women |
| No ID, ASD or ADHD | .25 (.25-.25) | .10 (.10-.10) | .07 (.06-.07) | .01 (.01-.01) | .07 (.07-.07) | .02 (.02-.02) |
| Mild ID only | .27 (.25-.28) | .11 (.10-.13) | .09 (.08-.10) | .03 (.02-.03) | .08 (.06-.11) | .07 (.06-.09) |
| Moderate/severe ID only | .10 (.08-.12) | .05 (.04-.07) | .05 (.04-.07) | .02 (.01-.04) | .02 (.01-.03) | .05 (.03-.07) |
| Mild ID + ASD | .18 (.15-.23) | .13 (.03-.18) | .09 (.06-.12) | .09 (.05-.14) | .05 (.03-.08) | .07 (.05-.10) |
| Moderate/severe ID + ASD | .08 (.04-.16) | .03 (.01-.06) | .04 (.02-.06) | .01 (.00-.02) | .01 (.00-.02) | .01 (.01-.04) |
| Mild ID + ADHD | .56 (.48-.64) | .31 (.26-.36) | .30 (.26-.35) | .12 (.08-.17) | .16 (.12-.20) | .18 (.15-.23) |
| Moderate/severe ID + ADHD | .39 (.32-.47) | .31 (.22-.42) | .27 (.20-.35) | .15 (.08-.27) | .14 (.09-.21) | .22 (.12-.37) |

ID, intellectual disability; ASD, autism spectrum disorder; ADHD, attention deficit hyperactivity disorder

Cumulative incidence estimated as 1 – Kaplan-Meier estimate of the survival function under the assumption of no competing risks.

**Supplementary Table 5.** Cumulative incidence (95% confidence interval) of sexual criminal convictions and sexual assault victimization by age 34 in people with mild and moderate/severe ID, stratified by comorbid ASD and ADHD

|  | **Sexual conviction** | | **Sexual assault** | |
| --- | --- | --- | --- | --- |
|  | Men | Women | Men | Women |
| No ID, ASD or ADHD | .004 (.004-.004) | NA | .000 (.000-.000) | .001 (.001-.001) |
| Mild ID only | .023 (.017-.030) | .001 (.000-.003) | .002 (.001-.003) | .057 (.047-.068) |
| Moderate/severe ID only | .007 (.003-.016) | NA | .002 (.001-.009) | .033 (.019-.058) |
| Mild ID + ASD | .020 (.011-.034) | NA | NA | .056 (.036-.086) |
| Moderate/severe ID + ASD | .004 (.001-.012) | NA | .001 (.000-.009) | .007 (.002-.032) |
| Mild ID + ADHD | .109 (.029-.364) | NA | .003 (.001-.009) | .118 (.086-.159) |
| Moderate/severe ID + ADHD | .042 (.021-.084) | NA | NA | .224 (.104-.445) |

ID, intellectual disability; ASD, autism spectrum disorder; ADHD, attention deficit hyperactivity disorder; NA, not applicable (could not be estimated)

Cumulative incidence estimated as 1 – Kaplan-Meier estimate of the survival function under the assumption of no competing risks.

**Supplementary Table 6.** Cox proportional hazard ratios (95% confidence intervals) for any criminal convictions, violent criminal convictions, and any assault victimization associated with mild and moderate/severe ID, stratified by comorbid ASD and ADHD

|  | **Any conviction** | | **Violent conviction** | | **Any assault** | |
| --- | --- | --- | --- | --- | --- | --- |
|  | Men | Women | Men | Women | Men | Women |
| **No ID, ASD or ADHD (ref.)** | 1 | 1 | 1 | 1 | 1 | 1 |
| Mild ID only | .93 (.88, .99) | .99 (.89, 1.10) | 1.07 (.96, 1.19) | 1.77 (1.44, 2.18) | .86 (.76, .97) | 2.51 (2.20, 2.87) |
| Moderate/severe ID only | .30 (.26, .36) | .42 (.32, .55) | .56 (.43, .72) | 1.29 (.85, 1.96) | .29 (.20, .42) | 1.40 (1.03, 1.90) |
| Mild ID + ASD | .63 (.52, .76) | 1.02 (.73, 1.42) | 1.06 (.79, 1.42) | 4.59 (2.96, 7.12) | .45 (.29, .70) | 3.03 (2.03, 4.53) |
| Moderate/severe ID + ASD | .16 (.12, .23) | .18 (.09, .38) | .36 (.22, .58) | .64 (.21, 1.99) | .09 (.03, .24) | .59 (.25, 1.42) |
| Mild ID + ADHD | 2.51 (2.29, 2.75) | 3.08 (2.58, 3.68) | 4.43 (3.91, 5.03) | 6.31 (4.56, 8.71) | 2.02 (1.68, 2.42) | 7.34 (5.83, 9.22) |
| Moderate/severe ID + ADHD | 1.71 (1.36, 2.14) | 3.32 (2.28, 4.84) | 3.82 (2.88, 5.07) | 10.4 (5.93, 18.4) | 2.03 (1.37, 3.01) | 7.72 (4.65, 12.8) |

ID, intellectual disability; ASD, autism spectrum disorder; ADHD, attention deficit hyperactivity disorder; NA, not applicable (could not be estimated)

**Supplementary Table 7.** Cox proportional hazard ratios (95% confidence intervals) for sexual criminal convictions sexual assault victimization associated with mild and moderate/severe ID, stratified by comorbid ASD and ADHD

|  | **Sexual conviction** | | **Sexual assault** | |
| --- | --- | --- | --- | --- |
|  | Men | Women | Men | Women |
| **No ID, ASD or ADHD (ref.)** | 1 | 1 | 1 | 1 |
| Mild ID only | 4.86 (3.86, 6.11) | 9.54 (2.22, 41.0) | 12.7 (5.71, 28.2) | 5.02 (4.30, 5.87) |
| Moderate/severe ID only | 1.29 (.61, 2.70) | NA | 10.8 (2.63, 44.5) | 2.58 (1.79, 3.72) |
| Mild ID + ASD | 5.96 (3.45, 10.3) | NA | NA | 6.40 (4.13, 9.94) |
| Moderate/severe ID + ASD | 1.27 (.41, 3.95) | NA | 9.63 (1.32, 70.5) | .62 (.15, 2.46) |
| Mild ID + ADHD | 9.39 (6.54, 13.5) | NA | 22.0 (6.79, 71.2) | 11.0 (8.26, 14.8) |
| Moderate/severe ID + ADHD | 11.0 (5.46, 22.1) | NA | NA | 17.1 (9.93, 29.5) |

ID, intellectual disability; ASD, autism spectrum disorder; ADHD, attention deficit hyperactivity disorder; NA, not applicable (could not be estimated)

**Supplementary Table 8.** Cox proportional hazard ratios (95% confidence intervals) for any, violent, and sexual crime suspicions associated with mild and moderate/severe ID, stratified by comorbid ASD and ADHD in population and within-family analyses

|  | **Population analyses** | | | | | |
| --- | --- | --- | --- | --- | --- | --- |
|  | **Any suspicion** | | **Violent suspicion** | | **Sexual suspicion** | |
|  | Men | Women | Men | Women | Men | Women |
| **No ID, ASD or ADHD (ref.)** | 1 | 1 | 1 | 1 | 1 | 1 |
| Mild ID only | 1.10 (1.04, 1.16) | 1.32 (1.22, 1.43) | 1.20 (1.12, 1.30) | 1.96 (1.75, 2.20) | 4.07 (3.58, 4.62) | 7.21 (3.52, 14.8) |
| Moderate/severe ID only | .45 (.39, .51) | .53 (.43, .66) | .70 (.59, .82) | 1.00 (.76, 1.32) | 1.72 (1.24, 2.39) | 5.28 (1.30, 21.3) |
| Mild ID + ASD | .81 (.70, .95) | 1.39 (1.08, 1.80) | 1.19 (.98, 1.45) | 3.14 (2.30, 4.28) | 3.01 (2.05, 4.43) | 19.7 (4.86, 79.5) |
| Moderate/severe ID + ASD | .40 (.32, .49) | .48 (.32, .73) | .68 (.53, .86) | 1.20 (.75, 1.94) | 1.40 (.81, 2.42) | NA |
| Mild ID + ADHD | 2.82 (2.60, 3.06) | 3.83 (3.30, 4.43) | 4.02 (3.64, 4.43) | 6.16 (5.06, 7.50) | 9.71 (8.08, 11.7) | 22.8 (7.24, 72.1) |
| Moderate/severe ID + ADHD | 2.09 (1.72, 2.54) | 3.86 (2.80, 5.32) | 3.16 (2.51, 3.96) | 8.20 (5.58, 12.0) | 8.14 (5.35, 12.4) | 68.4 (16.9, 277) |
|  | **Within-family analyses** | | | | | |
|  | **Any suspicion** | | **Violent suspicion** | | **Sexual suspicion** | |
|  | Men | Women | Men | Women | Men | Women |
| **No ID, ASD or ADHD (ref.)** | 1 | 1 | 1 | 1 | 1 | 1 |
| Mild ID only | .81 (.70, .93) | .98 (.80, 1.22) | .87 (.72, 1.05) | 1.27 (.91, 1.77) | 2.47 (1.67, 3.67) | .89 (.04, 19.9) |
| Moderate/severe ID only | .36 (.27, .49) | .39 (.24, .63) | .53 (.36, .76) | .90 (.45, 1.81) | 1.19 (.56, 2.52) | NA |
| Mild ID + ASD | .49 (.32, .75) | 1.51 (.78, 2.91) | .57 (.32, .99) | 4.59 (1.61, 13.1) | .99 (.26, 3.70) | NA |
| Moderate/severe ID + ASD | .36 (.22, .57) | .31 (.12, .85) | .59 (.34, 1.03) | .87 (.24, 3.15) | .88 (.28, 2.78) | NA |
| Mild ID + ADHD | 1.77 (1.35, 2.31) | 1.86 (1.15, 3.03) | 2.15 (1.56, 2.96) | 2.20 (1.13, 4.27) | 5.57 (2.62, 11.8) | NA |
| Moderate/severe ID + ADHD | 1.38 (.75, 2.54) | 1.84 (.59, 5.70) | 1.87 (.90, 3.87) | 1.71 (.36, 8.10) | 4.70 (1.13, 19.6) | NA |

ID, intellectual disability; ASD, autism spectrum disorder; ADHD, attention deficit hyperactivity disorder; NA, not applicable (could not be estimated)

**Supplementary Table 9.** Cox proportional hazard ratios (95% confidence intervals) for criminal convictions associated with mild and moderate/severe ID, stratified by comorbid ASD and ADHD in within-family analyses

|  | **Any conviction** | | **Violent conviction** | | **Sexual conviction** | |
| --- | --- | --- | --- | --- | --- | --- |
|  | Men | Women | Men | Women | Men | Women |
| **No ID, ASD or ADHD (ref.)** | 1 | 1 | 1 | 1 | 1 | 1 |
| Mild ID only | .74 (.63, .86) | .85 (.66, 1.10) | .67 (.51, .88) | 1.21 (.71, 2.04) | 2.76 (1.37, 5.56) | 9.54 (2.22, 41.0) |
| Moderate/severe ID only | .27 (.19, .39) | .32 (.17, .58) | .37 (.20, .66) | 2.37 (.63, 8.89) | .48 (.09, 2.55) | NA |
| Mild ID + ASD | .50 (.31, .80) | .75 (.31, 1.81) | .83 (.40, 1.73) | 2.59 (.66, 10.1) | .63 (.10, 4.14) | NA |
| Moderate/severe ID + ASD | .04 (.01, .16) | .07 (.01, .56) | .06 (.01, .47) | .48 (.05, 4.75) | 2.09 (.12, 37.4) | NA |
| Mild ID + ADHD | 1.49 (1.13, 1.97) | 2.58 (1.41, 4.71) | 2.57 (1.68, 3.93) | 2.09 (.64, 6.85) | 4.63 (1.36, 15.8) | NA |
| Moderate/severe ID + ADHD | 1.33 (.67, 2.63) | .76 (.21, 2.73) | 5.92 (1.85, 18.9) | 1.70 (.22, 13.4) | 10.2 (.90, 115) | NA |

ID, intellectual disability; ASD, autism spectrum disorder; ADHD, attention deficit hyperactivity disorder; NA, not applicable (could not be estimated)

**Supplementary Table 10.** Cox proportional hazard ratios (95% confidence intervals) for assault victimization associated with mild and moderate/severe ID, stratified by comorbid ASD and ADHD in within-family analyses

|  | **Any assault** | | **Sexual assault** | |
| --- | --- | --- | --- | --- |
|  | Men | Women | Men | Women |
| **No ID, ASD or ADHD (ref.)** | 1 | 1 | 1 | 1 |
| Mild ID only | .70 (.52, .96) | 1.82 (1.18, 2.80) | NA | 3.38 (1.83, 6.25) |
| Moderate/severe ID only | .37 (.18, .76) | .88 (.41, 1.92) | NA | .95 (.34, 2.68) |
| Mild ID + ASD | .34 (.09, 1.27) | .58 (.20, 1.69) | NA | .95 (.23, 3.99) |
| Moderate/severe ID + ASD | .10 (.01, .76) | .71 (.09, 5.75) | NA | 1.23 (.05, 31.5) |
| Mild ID + ADHD | .80 (.46, 1.38) | 7.26 (2.06, 25.5) | NA | 15.8 (1.95, 128) |
| Moderate/severe ID + ADHD | .74 (.20, 2.70) | 4.63 (.42, 51.3) | NA | NA |

ID, intellectual disability; ASD, autism spectrum disorder; ADHD, attention deficit hyperactivity disorder; NA, not applicable (could not be estimated)

**Supplementary Table 11.** Cox proportional hazard ratios (95% confidence intervals) for any, violent, and sexual crime convictions and assault victimization associated with mild and moderate/severe ID as defined by graduation from a special school for pupils with ID (HURPID) and having a diagnosis of mental retardation (NPR), stratified by comorbid ASD and ADHD

|  | **HURPID data** | | | | | | | |
| --- | --- | --- | --- | --- | --- | --- | --- | --- |
|  | **Any conviction** | | **Violent conviction** | | **Sexual conviction** | | **Assault victimization** | |
|  | Men | Women | Men | Women | Men | Women | Men | Women |
| **No ID, ASD or ADHD (ref.)** | 1 | 1 | 1 | 1 | 1 | 1 | 1 | 1 |
| Mild ID only | .76  (.71, .81) | .71  (.62, .81) | .78  (.69, .89) | 1.16  (.88, 1.51) | 3.49  (2.66, 4.60) | 5.28  (.71, 39.4) | .73  (.64, .85) | 1.92  (1.64, 2.26) |
| Moderate/severe ID only | .01  (.001, .07) | .03  (.005, .23) | NA | NA | NA | NA | .09  (.02, .37) | NA |
| Mild ID + ASD | .42  (.33, .53) | .29  (.14, .57) | .67  (.45, .98) | 1.16  (.43, 3.09) | 3.51  (1.67, 7.38) | NA | .29  (.16, .52) | 2.43  (1.46, 4.02) |
| Moderate/severe ID + ASD | NA | .10  (.01, .72) | NA | NA | NA | NA | NA | .44  (.06, 3.11) |
| Mild ID + ADHD | 1.69  (1.48, 1.93) | 1.75  (1.26, 2.44) | 2.46  (2.00, 3.02) | 3.49  (1.88, 6.49) | 5.74  (3.24, 10.2) | NA | 1.18  (.87, 1.59) | 5.00  (3.38, 7.41) |
| Moderate/severe ID + ADHD | NA | NA | NA | NA | NA | NA | NA | NA |
|  | **NPR data** | | | | | | | |
|  | **Any conviction** | | **Violent conviction** | | **Sexual conviction** | | **Assault victimization** | |
|  | Men | Women | Men | Women | Men | Women | Men | Women |
| **No ID, ASD or ADHD (ref.)** | 1 | 1 | 1 | 1 | 1 | 1 | 1 | 1 |
| Mild ID only | 1.14  (1.04, 1.25) | 1.31  (1.14, 1.51) | 1.65  (1.42, 1.91) | 2.75  (2.12, 3.56) | 6.74  (4.86, 9.34) | 11.5  (1.54, 86.2) | 1.00  (.82, 1.22) | 3.42  (2.86, 4.08) |
| Moderate/severe ID only | .34  (.28, .40) | .46  (.36, .60) | .61  (.47, .79) | 1.41  (.93, 2.14) | 1.37  (.65, 2.88) | NA | .31  (.21, .46) | 1.53  (1.12, 2.08) |
| Mild ID + ASD | .75  (.59, .94) | 1.29  (.91, 1.84) | 1.40  (1.00, 1.96) | 5.84  (3.68, 9.28) | 6.34  (3.17, 12.7) | NA | .53  (.31, .92) | 3.43  (2.19, 5.38) |
| Moderate/severe ID + ASD | .17  (.12, .25) | .20  (.09, .41) | .38  (.24, .62) | .69  (.22, 2.13) | 1.34  (.43, 4.16) | NA | .09  (.04, .25) | .63  (.26, 1.51) |
| Mild ID + ADHD | 2.63  (2.37, 2.91) | 3.38  (2.80, 4.06) | 4.85  (4.22, 5.58) | 6.77  (4.83, 9.48) | 10.8  (7.32, 15.9) | NA | 2.15  (1.75, 2.63) | 8.02  (6.33, 10.2) |
| Moderate/severe ID + ADHD | 1.73  (1.38, 2.16) | 3.36  (2.30, 4.90) | 3.81  (2.87, 5.06) | 10.47  (5.94, 18.5) | 11.0  (5.49, 22.2) | NA | 2.04  (1.38, 3.02) | 7.76  (4.67, 12.9) |

ID, intellectual disability; ASD, autism spectrum disorder; ADHD, attention deficit hyperactivity disorder; NA, not applicable (could not be estimated); HURPID, Halmstad University Register on Pupils with Intellectual Disability; NPR, National Patient Register

**Supplementary Table 12.** Cox proportional hazard ratios (95% confidence intervals) for assault victimization associated with mild and moderate/severe ID stratified by comorbid ASD and ADHD, excluding those with assault victimization predating ID registration (n=333)

|  | **Any assault** | | **Sexual assault** | |
| --- | --- | --- | --- | --- |
|  | Men | Women | Men | Women |
| **No ID, ASD or ADHD (ref.)** | 1 | 1 | 1 | 1 |
| Mild ID only | .55 (.47, .64) | 1.50 (1.26, 1.78) | 3.62 (.88, 15.0) | 2.95 (2.41, 3.60) |
| Moderate/severe ID only | .21 (.13, .32) | .92 (.63, 1.35) | 10.8 (2.62, 44.5) | 1.87 (1.22, 2.87) |
| Mild ID + ASD | .35 (.21, .59) | 1.66 (.97, 2.87) | NA | 3.57 (1.97, 6.45) |
| Moderate/severe ID + ASD | .07 (.02, .21) | .47 (.18, 1.26) | 9.44 (1.28, 69.5) | .62 (.15, 2.46) |
| Mild ID + ADHD | 1.26 (1.00, 1.59) | 4.66 (3.49, 6.23) | 21.6 (6.67, 70.2) | 7.04 (4.88, 10.1) |
| Moderate/severe ID + ADHD | 1.48 (.93, 2.35) | 2.67 (1.11, 6.42) | NA | 6.76 (2.81, 16.3) |

ID, intellectual disability; ASD, autism spectrum disorder; ADHD, attention deficit hyperactivity disorder; NA, not applicable (could not be estimated)

**Supplementary Table 13.** Cox proportional hazard ratios (95% confidence intervals) for criminal convictions and assault victimization associated with mild and moderate/severe ID with comorbid ASD and ADHD

|  | **Any conviction** | | **Violent conviction** | | **Any assault** | |
| --- | --- | --- | --- | --- | --- | --- |
|  | Men | Women | Men | Women | Men | Women |
| **No ID, ASD or ADHD (ref.)** | 1 | 1 | 1 | 1 | 1 | 1 |
| Mild ID + ASD + ADHD | 2.15 (1.84, 2.52) | 3.04 (2.25, 4.12) | 4.13 (3.33, 5.14) | 11.88 (7.89, 17.9) | 1.64 (1.17, 2.30) | 5.07 (3.19, 8.05) |
| Moderate/severe ID + ASD + ADHD | .74 (.50, 1.09) | 1.87 (1.03, 3.37) | 2.07 (1.30, 3.29) | 6.48 (2.69, 15.6) | .80 (.38, 1.69) | 5.59 (2.80, 11.2) |
|  | **Sexual conviction** | | **Sexual assault** | |  |  |
|  | Men | Women | Men | Women |  |  |
| **No ID, ASD or ADHD (ref.)** |  |  |  |  |  |  |
| Mild ID + ASD + ADHD | 14.4 (8.76, 23.5) | NA | 41.8 (10.1, 172) | 7.59 (4.20, 13.7) |  |  |
| Moderate/severe ID + ASD + ADHD | 4.42 (1.10, 17.7) | NA | NA | 12.4 (5.89, 26.0) |  |  |

ID, intellectual disability; ASD, autism spectrum disorder; ADHD, attention deficit hyperactivity disorder; NA, not applicable (could not be estimated)

**Supplementary Figure 1.** Cumulative incidence (estimated as 1 – the Kaplan-Meier estimate of the survival function under the assumption of no competing risks) of sexual crime convictions in men and sexual assault victimization in women with mild ID, stratified by comorbid ASD and ADHD.


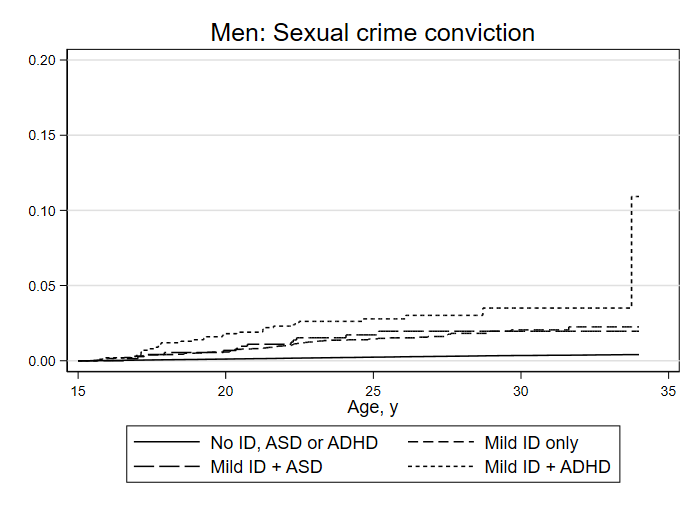

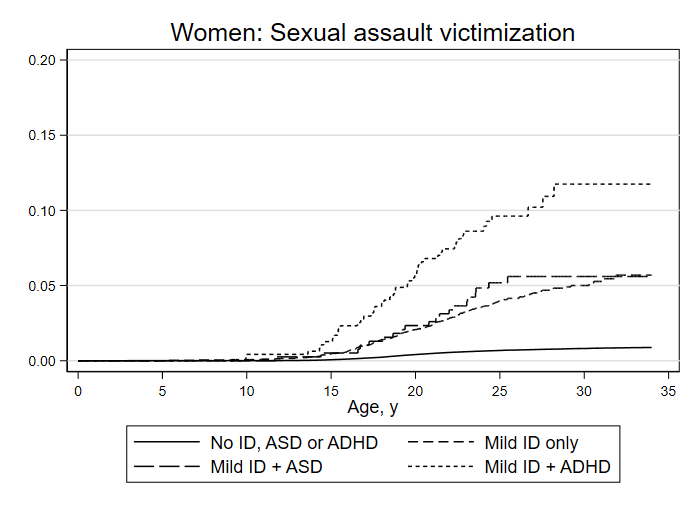


ID, intellectual disability; ASD, autism spectrum disorder; ADHD, attention deficit hyperactivity disorder
